# The Structural Evolution of Recrystallized Asymmetric SiC Membranes for High-Performance Oily Wastewater Treatment

**DOI:** 10.3390/membranes16060213

**Published:** 2026-06-21

**Authors:** Muhammad Shoaib Anwar, Jang-Hoon Ha, Jongman Lee, Hong Joo Lee, In-Hyuck Song

**Affiliations:** Nanomaterials Research Division, Korea Institute of Materials Science (KIMS), 797 Changwon-daero, Seongsan-gu, Changwon-si 51508, Gyeongsangnam-do, Republic of Korea; drmsa789@gmail.com (M.S.A.);

**Keywords:** asymmetric SiC membrane, microfiltration (MF), pore structure optimization, oil-in-water (O/W) separation, water flux recovery

## Abstract

Asymmetric SiC membranes with surface pore sizes ranging from 0.12 to 0.31 μm at a constant open porosity of approximately 42% were fabricated by dip-coating SiC support followed by sintering from 1700 to 2000 °C. The effect of membrane structural constants (hydraulic resistance (k_1_), pore size exponent (k_2_), and shape factor (k_3_)) on PWP were evaluated by comparing the symmetric and asymmetric structures. In addition, the experimentally determined values of PWP were quantitatively analyzed by comparing with theoretically predicted values obtained using the Kozeny–Carman (K–C) and Hagen–Poiseuille (H–P) models. Despite having a smaller pore size, the asymmetric membranes exhibited high PWP (1257-3883 LMH) due to decreased flow resistance (low k_1_), enhanced pore size effect (high k_2_), and improved flow network (high k_3_) as compared to symmetric membranes. The hydrophilicity of the prepared membranes improved remarkably, with increasing average surface roughness (102.3 nm to 161.0 nm) due to an increase in pore size, which also caused a decrease in water contact angle (WCA) from approximately 27.44° to 21.67° with increasing sintering temperature (1700–2000 °C). Furthermore, the prepared membrane separation performance was found to be affected by its pore size, and the 1900 °C sintered SiC membrane showed optimal gradient profile and pore structure, demonstrating its practical reusability and scalability for O/W wastewater treatment.

## 1. Introduction

The rising demand for clean water and effective wastewater handling has become a major global concern owing to the increasing world population and rapid industrialization [1,2,3,4,5]. Therefore, exploring new freshwater reserves and developing new economical technologies to purify the existing wastewater are crucial to meet the clean water supply-and-demand requirements [6,7]. In particular, the discharge of oily wastewater from several industrial sectors, such as the food, oil and gas, and beverage sectors, contributes significantly to environmental pollution, which causes severe public health issues [2,8,9,10]. Among existing water treatment technologies, membrane technology offers the advantage of low energy consumption and high process efficiency without the need for hazardous chemicals [6,11,12]. Membrane technologies based on ceramic membranes in particular can be effectively utilized for treating wastewater under harsh conditions owing to their superior separation performance and long-lasting structural integrity compared to polymeric membranes [3,13,14]. Although alumina (Al_2_O_3_) is the most commonly researched ceramic material owing to its low processing cost, pure SiC membranes, exhibiting excellent thermal, mechanical, and chemical stability even at relatively higher water fluxes, have emerged as suitable candidates for treating wastewater [15,16,17,18,19,20,21]. SiC membranes can be manufactured into a single-layer (symmetric) or a multilayer (asymmetric) structure by adopting a suitable processing technique.

Asymmetric SiC membranes are usually processed by depositing a thin SiC layer onto a suitable macro-porous support using chemical vapor deposition (CVD) [22,23], polymer-derived ceramic precursors (PDCs) [24,25], and fine particles [16,26]. CVD-coated membranes are relatively dense and expensive, leading to lower permeability values and higher fabrication costs, respectively. The use of PDCs offers advantages in compositional design and pore size control; however, low ceramic yields and high synthesis costs are major barriers to industrial scalability. In contrast, SiC membranes deposited using dip-, spin-, or spray-coating of fine-sized particles are relatively cost-effective, which permits scalability. Although some previous studies have reported on the fabrication of pure SiC membranes by coating fine-sized particles [15,21,26,27,28,29,30], their use and optimization in oil-in-water (O/W) separation, along with membrane cleaning, is still underexplored. In addition, reported studies have focused on single-layer symmetric SiC membranes [4,7,31], which face challenges in controlling pore size distribution and internal pore connectivity, leading to non-uniform flow resistance and reduced long-term performance. In contrast, multilayer asymmetric structures effectively address such limitations by introducing a finely controlled top layer with precise pore size while maintaining a highly permeable support layer. Despite SiC membranes offering advantages, a systematic study of their performance across various parameters, including water permeability, O/W separation, membrane fouling, and regeneration, is still lacking, particularly for multilayer asymmetric SiC membranes. Furthermore, symmetric membranes are simple to manufacture and can easily form a uniform structure; however, to ensure sufficient mechanical strength, they must be fabricated with a greater thickness, which limits their water permeability. In contrast, asymmetric membranes are fabricated by coating a thin separation layer onto a support with large pores, allowing them to maintain mechanical strength while reducing membrane thickness and thereby achieving higher permeability. Although these advantages have been qualitatively reported in several studies, a quantitative analysis of how much performance improvement an asymmetric structure offers over a symmetric structure under identical material and operating conditions has not yet been conducted. For example, the recent research on SiC membranes have emphasized their importance for separation applications. For instance, Q. Gu et al. [27] fabricated asymmetric SiC membranes with a thin (~20 μm) selective layer and narrow pore-size distribution, Y. Zhang et al. [32] fabricated asymmetric SiC via a foam–gel casting technique, W. Wei et al. [33] prepared gradient hierarchical SiC membranes, X. Hu et al. [34] and Z. Wang et al. [35] prepared surface-modified hydrophilic SiC membrane, B. Jing et al. [36] prepared a conductive Ni-coated SiC membrane for improved antifouling, and Y. Song et al. [37] prepared a hydrophobic SiC composite membrane; however, these previous reported studies lack a quantitative comparison of asymmetric and symmetric SiC membrane structure, which highlights the difference in permeability values.

In this context, this study presents the fabrication and systematic quantitative analysis of multilayer asymmetric pure SiC membranes for O/W separation. Unlike previous studies that primarily investigated symmetric SiC membranes, we fabricated asymmetric membranes via a single-step dip-coating process, enabling a thinner functional MF layer supported by a highly permeable macroporous structure. The pore size was tailored by varying the sintering temperature (1700–2000 °C), and the pure water permeability (PWP) was quantitatively evaluated using the Hagen–Poiseuille (H–P) and Kozeny–Carman (K–C) models to extract the key structural parameters—hydraulic resistance (k_1_), pore size exponent (k_2_), and shape factor (k_3_)—and to directly compare with conventional single-layer symmetric SiC membranes. This first-of-its-kind quantitative evaluation provides critical design guidelines for optimizing ceramic membrane architectures.

## 2. Materials and Methods

### 2.1. Fabrication of an Asymmetric SiC Membrane

SiC powders consisting of coarse α-SiC (~4.5 µm, Han Song Ltd., Shanghai, China) and fine α-SiC (15 wt%, ~0.55 µm, UF-15, H.C. Starck, Selb, Germany) particles were combined with B_4_C as a sintering additive (2 wt%, ˂10 µm, Sigma-Aldrich, St. Louis, MO, USA) and polyethylene glycol (PEG, 6 wt%, Mn = 4600, Sigma-Aldrich, St. Louis, MO, USA) as an organic binder to prepare disk-type porous support with a diameter of ~47.5 mm and a thickness of ~3.5 mm, as previously reported [19]. The target membranes on porous supports were deposited via dip-coating a prepared SiC slurry, which contained fine powder of α-SiC particles (~0.55 µm, 8 wt%, UF-15, H.C. Starck, Selb, Germany), deionized (DI) water (60 wt%), isopropyl alcohol (30 wt%, Samchun Chemicals, Pyeongtaek, Republic of Korea), polyvinyl alcohol (1 wt%, molecular weight: 31,000–50,000, Sigma-Aldrich, St. Louis, MO, USA), PEG (1 wt%, molecular weight: 1305–1595, Sigma-Aldrich, St. Louis, MO, USA), and 0.5 wt% of Darvan-CN (R.T. Vanderbilt Co., Norwalk, CT, USA) with respect to SiC powder. Details regarding optimizing the viscosity of the dip-coating SiC slurry are reported elsewhere [38]. A tabletop dip-coater (E-flex, Bucheon-si, Republic of Korea) was used to immerse the porous supports in the slurry for about 60 s, and subsequently, the coated supports were removed from the slurry at a speed of 1 mm/s. The coated supports were dried at room temperature (~25 °C) for 24 h, followed by high-temperature sintering at temperatures in the range of 1700–2000 °C for 2 h under an Ar atmosphere. The fabricated membranes on porous supports were assigned specific notations, as listed in Table 1. The numeric suffixes in the notation indicate the sintering temperature; for instance, M1700 stands for a SiC membrane sintered at 1700 °C.

### 2.2. Characterization of an Asymmetric SiC Membrane

Microstructural analysis of the sintered samples was conducted using a scanning electron microscope (SEM, JSM-5800, Jeol, Tokyo, Japan) coupled with energy dispersive spectroscopy (EDS). The surface roughness of the prepared SiC membranes was analyzed using atomic force microscopy (AFM, XE-100, Park Systems, Suwon-si, Republic of Korea). Phase analysis was carried out using an X-ray diffractometer (XRD, D/Max 2500 V/PC, Rigaku Corporation, Tokyo, Japan), and Rietveld analysis of the XRD data was performed using a previously reported methodology [20,39]. The pore size distribution was evaluated using a mercury porosimeter (Autopore IV 9510, Micromeritics, Norcross, GA, USA), and the open porosity was determined utilizing Archimedes’ method using DI water. The hydrophilicity of the membranes was assessed by measuring the water contact angle (WCA). A drop of water was placed on the membrane surface, and the WCA was measured using a goniometer (Phoenix-MT (A), SEO, Selangor D.E., Petaling Jaya, Malaysia).

A dead-end filtration system was used to evaluate the PWP and O/W separation performance of the fabricated membranes at a transmembrane pressure of 1 ± 0.2 bar. To ensure the statistical reliability and reproducibility of the experimental results, a minimum of three samples were tested for both the PWP and O/W filtration experiments. The correlation between PWP and pore structure was analyzed using the modified K–C and H–P equations [4,40], presented as Equations (1) and (2), respectively.

(1) $$J=\frac{\Delta p\cdot d_{m}^{k_{2}}\cdot \varepsilon^{3}}{k_{1}\cdot ({1-\varepsilon )}^{2}\cdot \mu \cdot L}$$

(2) $$J=\frac{\Delta p\cdot d_{m}^{2}\cdot \varepsilon^{2}}{{32\cdot k}_{3}\cdot \mu \cdot L}$$

where J, Δp, d_m_, ε, μ, and L represent pure water flux, transmembrane pressure, membrane pore size, open porosity, liquid viscosity, and membrane thickness, respectively. k_1_, k_2_, and k_3_ are undetermined model coefficients obtained by fitting the experimental data.

### 2.3. Performance Evaluation During O/W Emulsion Filtration

An O/W emulsion with a concentration of 1000 ppm and a measured average droplet size of 356.2 nm was prepared by adding vacuum pump oil (Neovac MR-200; Moresco Corporation, Kobe, Japan) to DI water, as reported previously [5], and is schematically shown in Figure S1. The separation performance of the fabricated membrane during the O/W emulsion test was assessed in four steps. Step 1 was designed to determine the stable baseline flux (J_p_) using DI water. Step 2 was designed to determine the permeation capability (J_1_) of the membrane in the O/W emulsion. Step 3 involved ultrasonic cleaning of the assessed membrane to remove the surface-adhered oil droplets using sodium dodecyl sulfate (SDS) solution (10 mM) and DI water. Step 4 involved permeation of DI water through the assessed membrane to acquire a stable flux of pure water (J_2_). The fouling ratio of reversible (R_rev_, expressed in percentage), irreversible (R_ir_, expressed in percentage), and flux recovery rate (FRR, expressed in percentage) were calculated by substituting the measured values of J_p_, J_1_, and J_2_ in Equations (3)–(5), respectively. The rejection rate (RR, expressed in percentage) in the filtration test was calculated by comparing the oil concentrations in the feed (C_f_) and permeate (C_p_) obtained after analyzing the UV–visible absorption data (in Equation (6)). UV–visible absorption measurements were performed using a spectrophotometer (Optizen pop-v, k-lab, Daejeon, Republic of Korea).

(3) $$R_{\mathrm{rev}}\left(\%\right)=\left(\frac{J_{2}-J_{1}}{J_{p}}\right)\cdot \;100$$

(4) $$R_{\mathrm{ir}}\left(\%\right)=\left(\frac{J_{p}-J_{2}}{J_{p}}\right)\cdot \;100$$

(5) $$\mathrm{FRR}\;\left(\%\right)=\left(\frac{J_{2}}{J_{p}}\right)\cdot \;100$$

(6) $$\mathrm{RR}\;\left(\%\right)=\left(\frac{C_{f}-{\;C}_{p}}{C_{f}}\right)\cdot \;100$$

### 2.4. Fouling Mechanism Study During O/W Emulsion Filtration

Membrane fouling during O/W filtration was investigated using Hermia models—cake filtration, intermediate pore-blocking, standard pore-blocking, or complete pore-blocking models. The linearized forms of the four fouling models in terms of membrane flux (J) as a function of time (t) are represented by Equations (7)–(10) for the cake filtration, intermediate pore-blocking, standard pore-blocking, and complete pore-blocking models, respectively [5,41,42,43]. In this study, the four fouling models were used to fit and analyze the experimental data. The correlation coefficient (R^2^) value was determined using linear regression analysis. The model with an R^2^ value close to 1 was regarded as the best fit, and the model was considered to have effectively captured the underlying mechanisms driving fouling.

(7) $${\;J}_{1}^{-2}=J_{2}^{-2}+k_{c}\cdot t$$

(8) $${\;J}_{1}^{-1}={\;J}_{2}^{-1\;}+{\;k}_{i}\cdot t$$

(9) $$J_{1}^{-0.5}=J_{2}^{-0.5}+{\;k}_{s}\cdot t$$

(10) $$\ln\left(J_{1}^{-1}\right)=\ln\left(J_{2}^{-1}\right)+k_{b}\cdot t$$

where J_1_, J_2_, and t represent the O/W emulsion flux, pure water flux, and filtration time, respectively. k_c_, k_i_, k_s_, and k_b_ are undetermined model coefficients obtained by fitting the experimental data.

## 3. Results and Discussion

### 3.1. Asymmetric Pure SiC Membrane; Microstructure Evaluation

**Figure 1:**
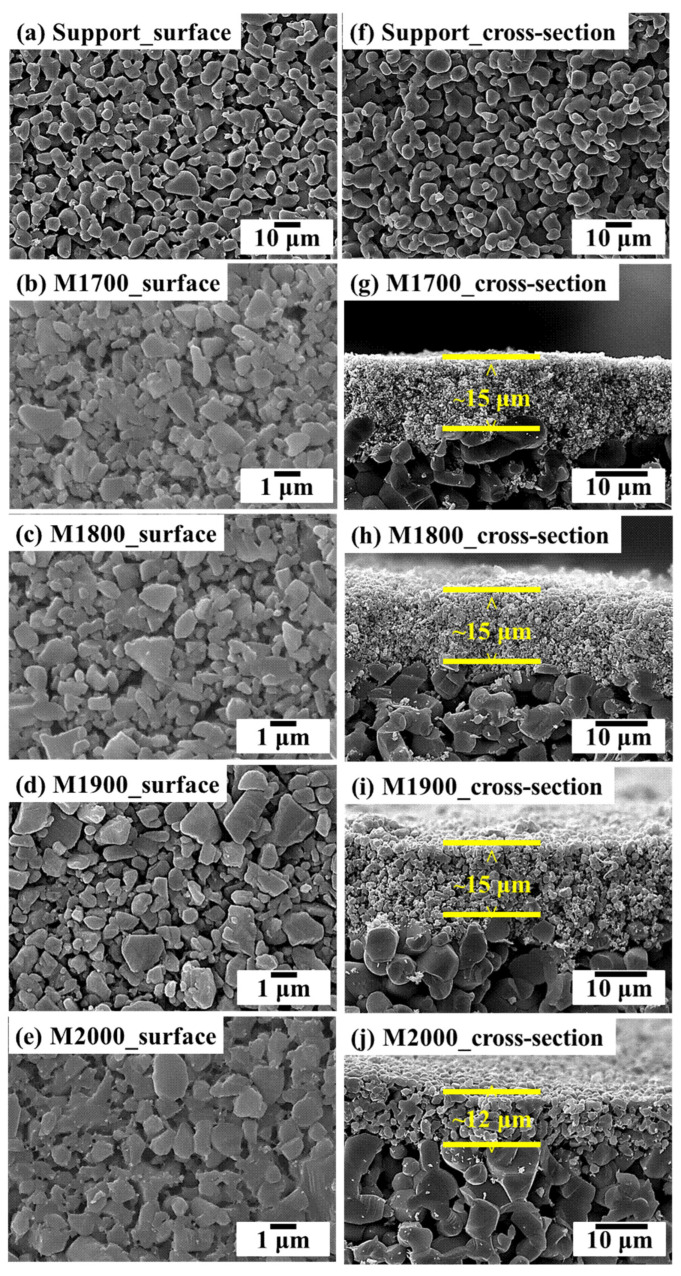
SEM image showing the surface (**a**–**e**) and fractured cross-sectional (**f**–**j**) morphologies of the SiC support and MF membranes.

A pure asymmetric SiC membrane was fabricated by dip-coating a macro-porous SiC support with sub-micron-sized SiC slurry, followed by high-temperature sintering (as schematically shown in Figure S2). The surface and fractured morphologies of the SiC support and coated MF membranes are shown in Figure 1.

**Figure 2:**
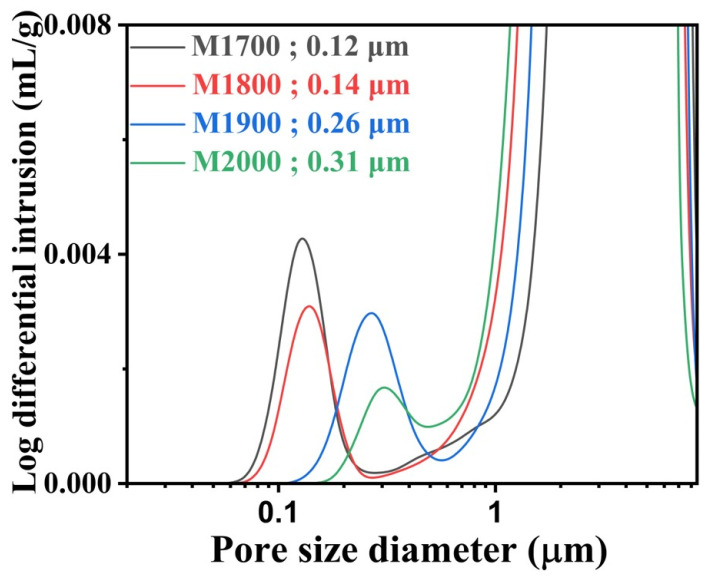
Pore size distribution of SiC MF membranes sintered from 1700 to 2000 °C.

The SEM images of the support (Figure 1a,f) show a well-connected macroporous structure with wide neck regions between the particles. These observed features can be attributed to high-temperature recrystallization processes [19,26], which are a combination of densification and structural refinement where fine particles merge into coarse grains, forming ‘neck regions’ that bridge the gap between individual particles. This phenomenon allows for the preparation of structures with high pore connectivity and open porosity, even with limited sintering additives, while simultaneously retaining the properties of the membranes under harsh conditions. Such macro-porous support structures ensure high water permeability owing to the reduced hydraulic resistance, which is important for the multilayer membrane structures. The SEM images of the surfaces (Figure 1b–e) and cross-sections (Figure 1g–j) of membranes sintered at different temperatures show crack-free membranes with well-defined boundaries and without any penetration to the macroporous supports. Slurry penetration into porous supports often occurs due to a mismatch between the viscosity of the coating slurry and surface properties, such as support pore size; in such cases, an intermediate layer is often used to prevent the slurry from penetrating too deeply into the support [28]. This study emphasizes that the need for depositing an intermediate layer can be avoided by fabricating a support with suitable surface properties. SEM analysis further reveals that the membrane particles tended to coarsen along with a decrease in the coating thickness (from ~15 μm to ~12 μm) with an increase in the sintering temperature from 1700 to 2000 °C. The membranes retained the thickness of ~15 μm up to a sintering temperature of 1900 °C; however, a further increase to 2000 °C caused the membrane thickness to decrease. This phenomenon is attributed to the Ostwald ripening and evaporation–condensation of relatively smaller particles [19,29], which also contributes to the increase in the shrinkage and pore size of the membrane, as also corroborated from the pore size analysis (Figure 2) of the membranes. This revealed that a maximum sintering temperature of 1900 °C was suitable to sinter the membranes to avoid excessive evaporation of fine particles, which reduced the effective thickness of the membrane. The average pore size values slowly increased from 0.12 to 0.31 μm (Figure 2) with the increase in the sintering temperature from 1700 to 2000 °C; however, the corresponding measured open porosities showed a value of ~42%, irrespective of the sintering temperature, which can be attributed to relatively low membrane thicknesses and highly connected open-pore structures within the membranes. In contrast to the prepared asymmetric SiC membrane, previous reports suggest that the open porosity of symmetric SiC membranes is largely affected by the sintering temperature [4] due to the complex porous structures of the membranes, which enhances the hydraulic resistance and reduces the permeability.

### 3.2. Asymmetric Pure SiC Membrane; Pure Water Permeability (PWP) Evaluation

**Figure 3:**
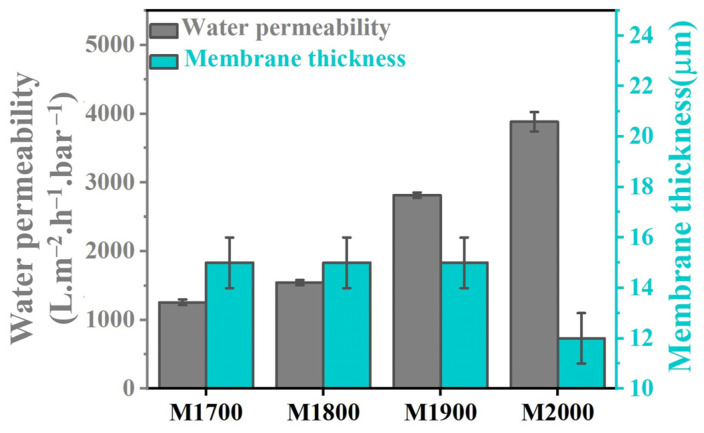
PWP comparison of the prepared SiC membranes sintered from 1700 to 2000 °C.

A lab-scale dead-end filtration system was developed to evaluate the permeability of the prepared membranes, as schematically shown in Figure S3. The effect of a change in sintering temperature on the resultant permeability is evident from Figure 3. The PWP largely increased from ~1256 L.m^−2^.h^−1^.bar^−1^ (LMH/bar) for M1700 to ~3883 LMH/bar for M2000, with an increase in corresponding pore size from 0.12 to 0.31 μm (Table 1). The decrease in the membrane thickness from 15 μm (M1700-M1900) to 12 μm (M2000) is also considered beneficial for improving the permeability owing to reduced hydraulic resistance; however, the change in the pore size was more prominent in affecting the permeability values. The general form of the Hagen–Poiseuille (H–P) equation [19,44] is often used to correlate the permeability and microstructure of the ceramic membrane, which is presented in Equation (11).

(11) $$J=\frac{\varepsilon r^{2}}{8\eta \tau }\times \frac{∆p}{∆x}$$

where J, Δp, r, ε, τ, η, and Δx represent the water flux, transmembrane pressure, pore size, porosity, tortuosity factor, viscosity of the fluid, and sample thickness, respectively. The values of Δp, ε, and η were kept constant, and Δx was slightly changed for M2000 only, which corroborated that the pore size of the membrane largely drove the change in permeability. As the connected pores in the SiC membranes are formed by the accumulation of coated particles, they are dependent on the size of the starting particles as well as the sintering temperature [19,30]. The SiC particle size in the coating slurry was kept constant; therefore, an increase in pore size can be largely attributed to the sintering temperature.

### 3.3. Quantitative Evaluation of Structural Parameters and Comparison Between Symmetric and Asymmetric Membranes

**Figure 4:**
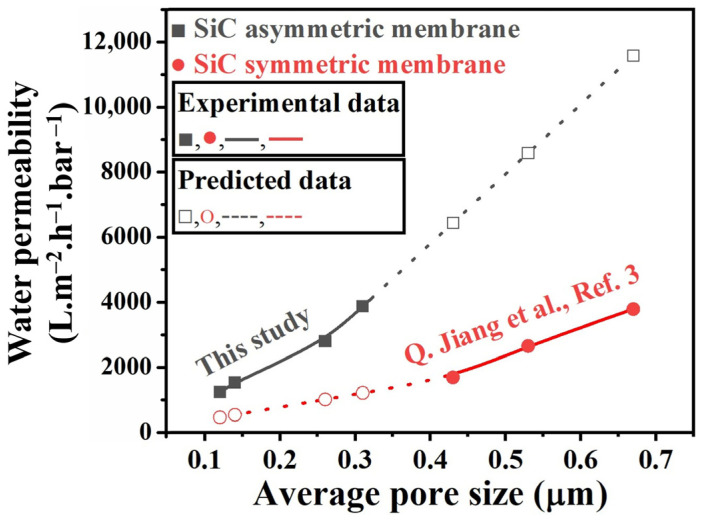
Comparison of the effect of pore size on the PWP of symmetric [3] and asymmetric SiC membranes.

The resultant permeability revealed that the prepared asymmetric SiC membranes, despite having significantly smaller pore sizes (0.12–0.31 μm), exhibited similar or even higher water permeabilities (1256–3883 LMH/bar) than those of their reported single-layered symmetric counterparts (1700–3800 LMH/bar) with pore sizes ranging from 0.43 to 0.67 μm and a porosity of 40% [4], as shown in Figure 4. Conventionally, a decrease in pore size leads to a decrease in water permeability due to increased hydraulic resistance. However, in our study, even with pore sizes reduced by more than half, the multilayered asymmetric SiC membranes exhibited unchanged or improved water permeability facilitated by the gradient porous structures, which primarily optimizes pore connectivity and reduces hydraulic resistance [45]. A key advantage of such a multilayered structure is its ability to demonstrate excellent mechanical stability even at a relatively lower overall membrane thickness. In single-layered structures, the thickness of the membrane must often be increased to ensure structural stability, which, in turn, increases transport resistance and decreases permeability. In contrast to this, in a multilayered membrane structure, the thin MF layer acts as the primary separation layer, whereas the support layer provides a porous structure, strength, and stability, resulting in higher permeability without compromising separation performance.

**Figure 5:**
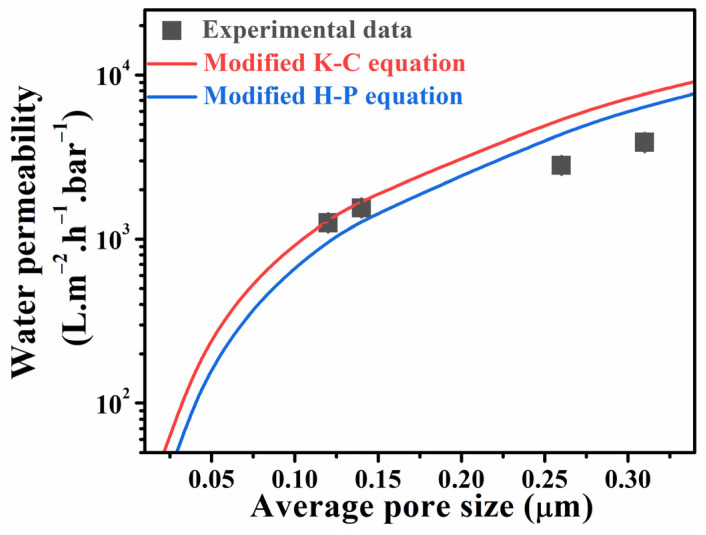
Experimental and theoretical PWP values of the fabricated SiC membranes. Theoretical PWP values were calculated using the Kozeny–Carman (K–C) and Hagen–Poiseuille (H–P) models.

**Figure 6:**
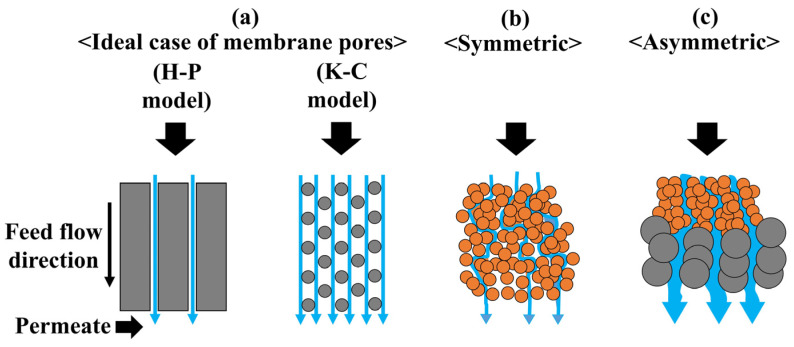
Schematic showing the effect of membrane structure on the water flow pattern. (**a**) Ideal case of membrane pore structures, (**b**) symmetric membrane pore structure, and (**c**) asymmetric membrane pore structure.

In addition, the experimentally derived PWP values were compared with those calculated using the modified K–C (Equation (1)) and H–P equations (Equation (2)) to correlate the effect of membrane pore structure with measured permeability; the resultant curves are shown in Figure 5, and the obtained model parameters are listed in Table 2. The correlation coefficient (R2) was higher than 0.99 in both calculated models, which validates the fitting models. Given that water permeability largely depends on the pore size at a constant porosity [46,47], a comparison of the data shown in Figure 5 indicates that the experimental values of PWP closely match the theoretically calculated values at smaller pore sizes. However, at higher pore sizes, a deviation between the experimental and theoretical values can be observed. The observed deviation between the experimental and theoretical values can be attributed to the complexities arising from variations in pore size, shape, and distribution within the membrane pore structure [46,47]. Such structural complexities often make it difficult to accurately predict the permeability values using a constant pore size in the theoretical model. Despite this, the results shown in Figure 5 reveal that the pore structure of the SiC membranes can be understood as a collection of interconnected pores, which, when viewed collectively, resemble a bundle of capillaries. The results of model-based analysis (Table 2) indicate that the permeability of ceramic membranes is influenced by several structural factors, including hydraulic resistance (k_1_), pore size exponent (k_2_), and shape factor (k_3_, such as particle size distribution and shape irregularity), as schematically shown in Figure 6. In the case of an ideal pore structure, as illustrated by the theoretical H-P and K-C models for perfectly cylindrical and uniformly distributed pores (Figure 6a), there is no hydraulic resistance, and the membrane structural parameters assume the ideal values as k_1_ = 1, k_2_ = 2, and k_3_ = 1; however, the actual pore structure (Figure 6b,c) deviates substantially from the ideal case. In actual membranes, k_1_ becomes larger when the internal pore network exhibits higher tortuosity or partial blockage, which increases the hydraulic resistance and reduces permeability; conversely, k_1_ decreases when the structure facilitates shorter and more direct flow paths, as in well-designed asymmetric architectures. k_2_, which reflects the sensitivity of permeability to pore size, may differ from the ideal value of 2 due to non-uniform pore size distributions, constricted pore throats, or irregular pore shapes. A higher k_2_ value indicates that the membrane’s flux is more sensitive to changes in pore size, which is often observed in asymmetric or thin selective layers where even minor variations in pore dimensions lead to significant differences in transport resistance. Conversely, a lower k_2_ value suggests that the membrane’s permeability is less dependent on pore size, typically corresponding to thicker or more homogeneous structures where transport is dominated by other resistances. The parameter k_3_ is a factor that reflects the microstructural characteristics of the membrane and can be expressed as the product of porosity and tortuosity. Therefore, a larger k_3_ value indicates that, at the same porosity, the flow paths are more tortuous, or, at the same tortuosity, the porosity is higher. In other words, a higher k_3_ means that the fluid travels along a longer path within the membrane or permeates through a relatively larger pore volume. These characteristics are particularly pronounced in asymmetric structures with multilayer architectures or a wide particle size distribution.

These deviations in k_1_, k_2_, and k_3_ from their ideal values directly capture how structural non-idealities in real membranes impact flow resistance, pore-size dependence, and geometric flow optimization. Compared with the symmetric membrane, the asymmetric membrane exhibits structural parameter values (k_1_, k_2_, and k_3_) that are all closer to their corresponding ideal-case values (Table 2). This indicates that, from a structural perspective, the asymmetric membrane more closely approaches the ideal pore architecture, which contributes to its superior hydraulic performance and optimized flow pathways. The substantial reduction in membrane thickness in the asymmetric design, together with the presence of large macro-pores in the support layer, significantly lowers the overall transport resistance for water molecules. In this configuration, the influence of surface pore size on permeability becomes more pronounced, as reflected by the higher k_2_ value. This indicates that even small variations in pore size produce a greater effect on water flux because the shorter transport distance amplifies the impact of the entrance pore characteristics. The higher k_3_ value predicted for the asymmetric membrane suggests more complex pore geometry compared to the ideal cylindrical case, arising from its gradient or multilayer porous structure. This complexity reflects factors such as a wider particle size distribution and irregular particle shapes, which modify flow convergence and divergence within the pores. In contrast, symmetric membranes lack such hierarchical structuring. While they tend to have more homogeneous pore geometry (lower k_3_) due to the absence of multiple layers and particle size variation, their substantially greater thickness imposes much higher hydraulic resistance (k_1_) and thus lower permeability. Overall, the model-derived structural constants clearly demonstrate that asymmetric membranes combine low hydraulic resistance (low k_1_), enhanced sensitivity of flux to surface pore size (high k_2_), and a flow network optimized by structural complexity (high k_3_). These characteristics enable asymmetric membranes to offer a more predictable and tunable design framework, providing a robust basis for the development of next-generation high-performance ceramic membrane systems. It should also be noted that the comparison between the present asymmetric SiC membrane and literature-reported symmetric SiC membranes in terms of structural constants (k_1_, k_2_, and k_3_) is subject to certain limitations because the membranes differ in fabrication route, pore characteristics, thickness, support configuration, and operating conditions. Therefore, the variations observed in the fitted parameters (k_1_, k_2_, and k_3_) is also affected by the membrane processing conditions along with membrane architecture. Nevertheless, the comparison provides a useful qualitative benchmark for assessing the potential benefits of the asymmetric structure on permeation.

### 3.4. Influence of Membrane Surface Characteristics on the Deviation of Structural Parameters

**Figure 7:**
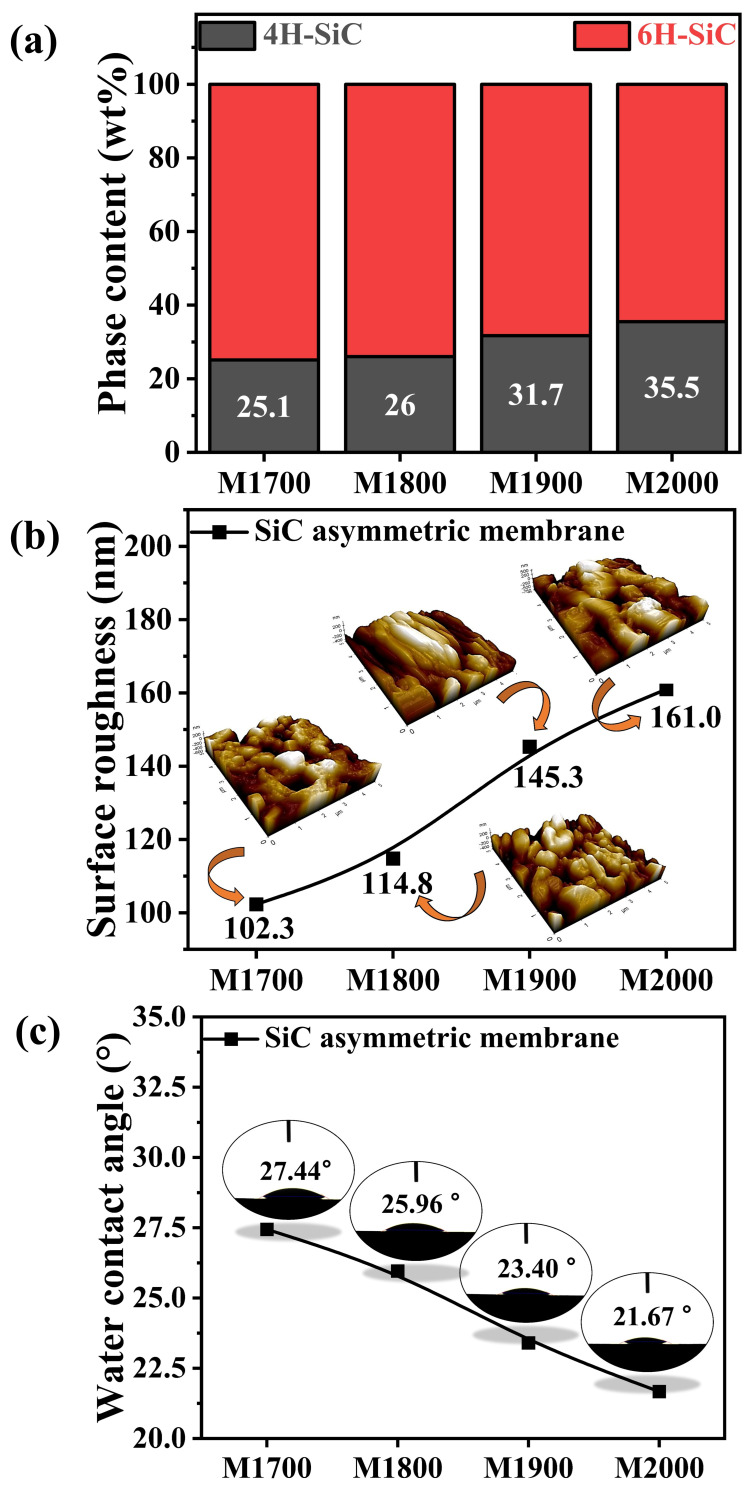
Properties of the prepared SiC membranes sintered from 1700 to 2000 °C. (**a**) Phase content, (**b**) surface roughness, and (**c**) instant WCA.

It is important to note that the determination of k_1_, k_2_, and k_3_ in this study considered only structural factors related to the internal pore geometry of the membranes, without incorporating surface property effects such as roughness, wettability, and surface energy. Therefore, the deviations observed in Figure 5 between the experimentally measured and theoretically predicted permeability values can be attributed to differences in surface characteristics, as evidenced in Figure 7. These surface property variations, induced by changes in sintering temperature, alter the effective water–membrane interactions and flow behavior at the pore entrances, which are not captured in the current theoretical models. The correlation of the change in the sintering temperature on membrane properties such as phase content, surface roughness and water contact angle (WCA) is evident from the results, as shown in Figure 7a, Figure 7b and Figure 7c, respectively. XRD patterns (Figure S4) showed the formation of a pure SiC membrane with a change in structural morphology from 6H-SiC polytype to 4H-SiC polytype with the rise in the sintering temperature, as shown in Table S1, which is attributed to the thermal stability of the 4H-SiC polytype at higher sintering temperatures [19,29]. The proportion of the 4H-SiC polytype gradually increases from 25.1 wt% to 35.5 wt% (Figure 7a) as the sintering temperature increases from 1700 to 2000 °C. This polytype transformation is accompanied by a change in the particle shape from spherical to relatively elongated, which affects properties such as pore size distribution, surface roughness, and WCA [19,26]. AFM was carried out to evaluate the surface morphology and roughness of the SiC membranes sintered at different temperatures, and the results are shown in Figure 7b. The AFM results show that the SiC grains grow and the average surface roughness increases from 102.3 nm to 161.0 nm as the sintering temperature increases from 1700 to 2000 °C due to an increase in the pore size of the membrane [4,16]. Similarly, the WCA of membranes slowly decreases from ~27.44° to ~21.67° (Figure 7c), whereas the water droplet penetration rate rapidly increases (Figure S5) as the sintering temperature increases from 1700 to 2000 °C, which indicates an improved hydrophilicity of the membrane. An increase in pore size facilitates capillary infiltration of water into the membrane surface, leading to a rapid decrease in WCA and contributing to enhanced permeability. The WCA of the membranes sintered at 1900 °C and 2000 °C decreases to nearly 0° within 5 s (Figure S5), indicating excellent hydrophilicity.

### 3.5. Asymmetric Pure SiC Membrane; O/W Separation Performance

**Figure 8:**
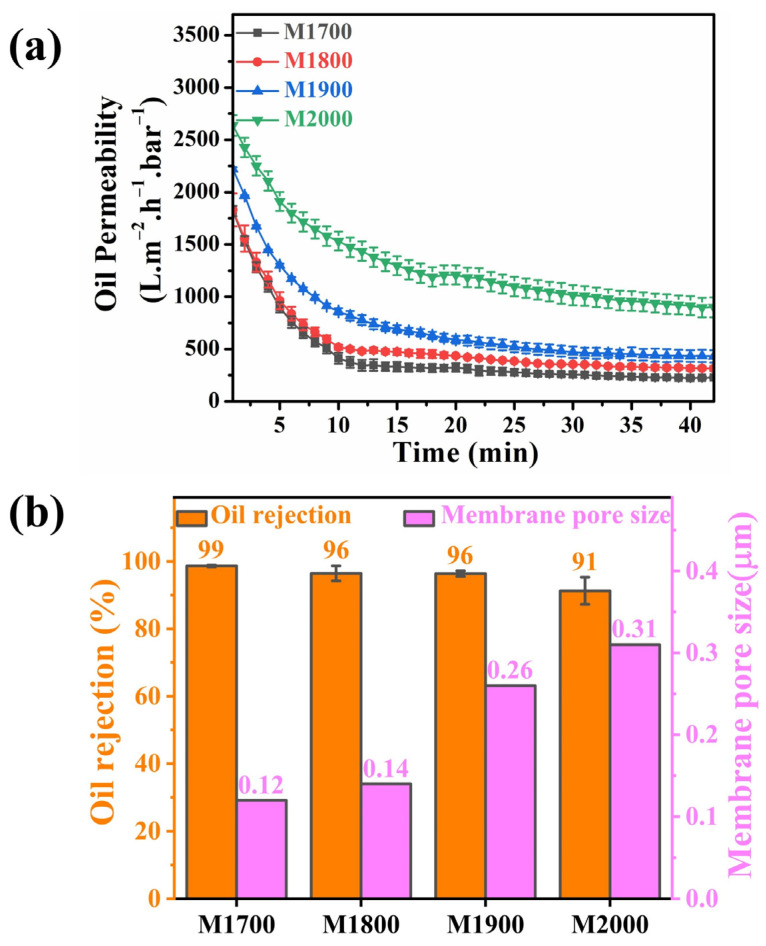
Effects of pore size on oil-in-water (O/W) emulsion (1000 ppm concentration) purification of SiC MF membranes sintered from 1700 to 2000 °C. (**a**) O/W emulsion permeability, (**b**) O/W emulsion rejection (%).

The effectiveness of the asymmetric SiC membranes in treating oily wastewater was tested using an O/W emulsion with a high concentration of 1000 ppm, and the obtained results are presented in Figure 8. The stable O/W emulsion permeability increases from 226 ± 15 LMH/bar to 905 ± 93 LMH/bar (Figure 8a), whereas the corresponding oil rejection decreases from approximately 99% to ~91% (Figure 8b) as the membrane pore size increases from ~0.12 μm to ~0.31 μm. An increase in the membrane pore size reduces the resistance to fluid flow; however, it compromises the effectiveness of filtering out unwanted impurities. As shown in Figure 8a, the permeability of the tested membranes in the highly concentrated O/W emulsion rapidly declined during the first 10 min of the test; however, the membranes exhibited a stable permeability with prolonged filtration time; this behavior of ceramic membranes is often observed in oil filtration tests. The observed behavior exhibited by the tested membranes can be attributed to fouling by oil droplets, leading to a decrease in permeability until a stable value is reached [48]. Despite an initial decline in permeability, the SiC membranes exhibited a highly stable flux under harsh conditions. The M1700 membrane exhibited a smaller mean pore size of 0.12 μm, which helped retain oil droplets through the pore size sieving effect and resulted in a rejection rate of nearly 99%. However, the M1700 membrane exhibited a relatively low permeability of 226 ± 15 LMH/bar. An increase in the membrane pore size to 0.31 μm, with an increase in the sintering temperature to 2000 °C, improved the permeability of the membrane to 905 ± 93 LMH/bar but reduced the rejection rate to 91%. In contrast, the M1900 membrane, with a high permeability of 433 ± 55 LMH/bar, a high rejection rate of 96%, and a suitable pore size distribution, effectively retained the oil droplets while demonstrating optimized permeability.

**Figure 9:**
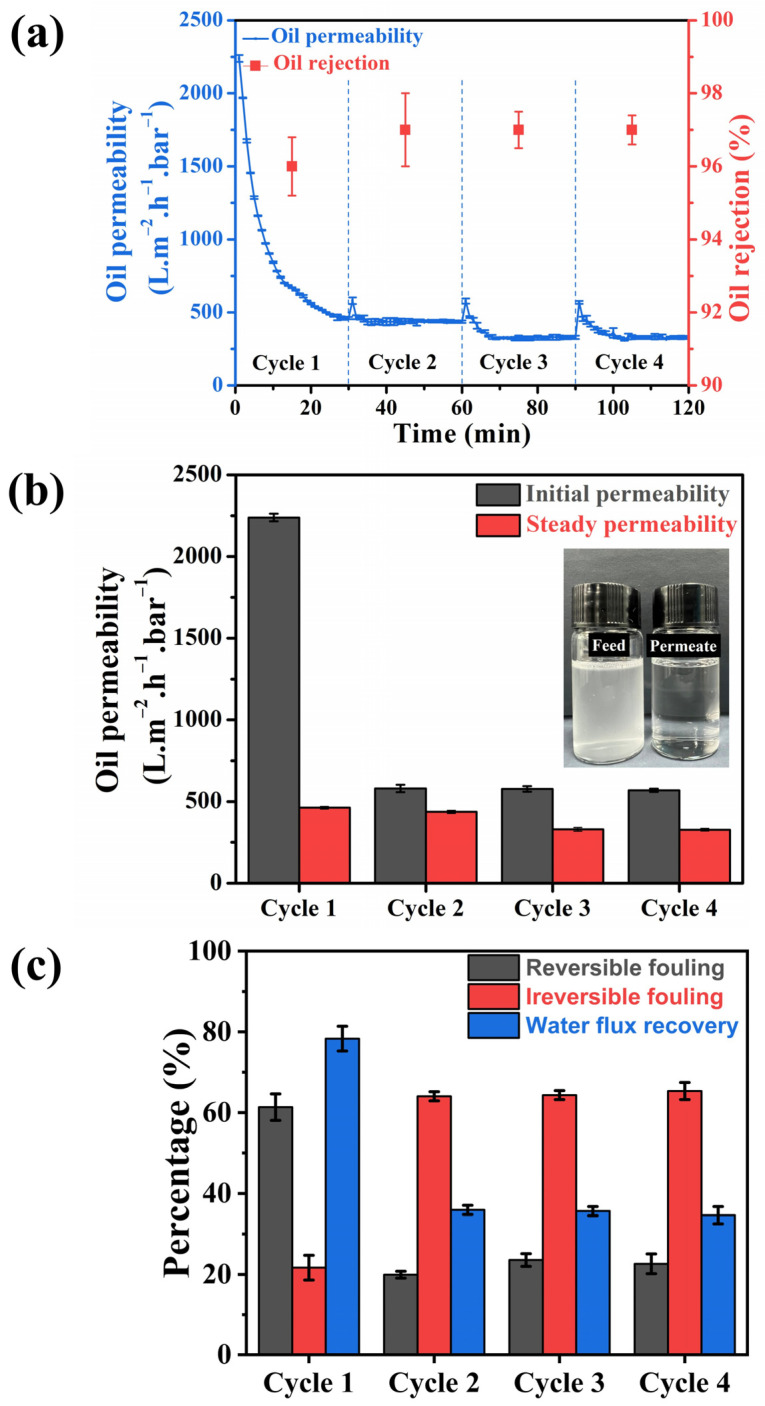
Performance of the SiC MF membrane sintered at 1900 °C in separating O/W emulsion (1000 ppm concentration) for four filtration cycles. O/W emulsion (**a**) permeability and oil rejection (%). (**b**) Comparison of initial and steady permeabilities. (**c**) Fouling ratios (reversible and irreversible) and water flux recovery.

The separation performance of the M1900 membrane under repeated cycles of O/W emulsion filtration was assessed, and the results are shown in Figure 9. The results presented in Figure 9a,b show that the initial permeability of the M1900 membrane rapidly decreased from 2239 ± 24 LMH/bar to a stable value of 433 ± 23 LMH/bar during the first cycle of the test. The permeability slightly recovered to 580 ± 23 LMH/bar after the surface cleaning step at the start of the second cycle, which is quite low as compared to that observed at the start of the test (2239 ± 24 LMH/bar). Notably, the M1900 membrane exhibited permeability values of 577 ± 18 LMH/bar and 569 ± 10 LMH/bar at the start of the third and fourth cycles, respectively.

The stable permeability values were determined to be 436 ± 9, 329 ± 11, and 327 ± 8 LMH/bar at the end of the second, third, and fourth cycles, respectively. The water flux recovery capacity decreased to 78 ± 3%, 36 ± 1%, 36 ± 1%, and 35 ± 2% of the initial value by the end of the second, third, and fourth cycles, respectively. The observed decrease in the initial permeability value can be attributed to rapid fouling of the membrane at the start of the test, as revealed by an increase in irreversible fouling of the membrane (Figure 9c). The irreversible fouling of the membrane rapidly increased to 64 ± 1% of the initial value by the end of the second cycle, after which the membrane exhibited more stable fouling and water flux recovery. During the O/W filtration tests, oil droplets often adhere to the surface of the membranes, resulting in blocking of the surface pores and increasing the intensity of the drag force on the incoming oil droplets, eventually increasing membrane fouling [5,49]. The membrane cleaning step does not completely remove the oil droplets, as also revealed by a lower recovery of water flux, making the membrane resistant to attaching additional oil droplets in subsequent cycles during the filtration test. Consequently, membranes often exhibit constant values of permeability, fouling, and water flux recovery capacity in subsequent filtration cycles.

**Figure 10:**
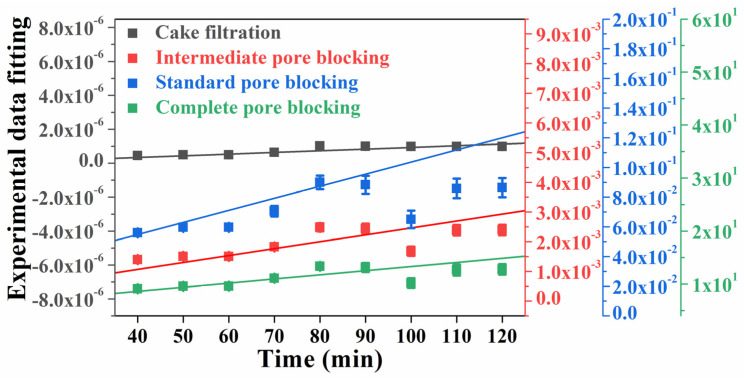
Experimental data fitting with Hermia (cake filtration, intermediate pore blocking, standard pore blocking, and complete pore blocking) models.

In this study, Hermia fouling models (as schematically shown in Figure S6) are used to predict the fouling mechanism of the SiC membrane, and the corresponding pore-blocking models are compared in Figure 10, with the fitting parameters listed in Table 3. The Hermia model fitting results indicate that the cake filtration model provided the highest correlation coefficient (R^2^ = 0.9805), closely followed by the intermediate pore-blocking model (R^2^ = 0.9798). Given the very small difference between these fitting coefficients, the results do not allow for an unequivocal distinction between the two fouling mechanisms based solely on statistical fitting. Rather, they suggest that both cake-layer formation and intermediate pore blocking may contribute to the overall fouling behavior during O/W emulsion filtration. The slightly higher correlation coefficient obtained for the cake filtration model is consistent with the dead-end filtration configuration employed in this study, where retained oil droplets progressively accumulate on the membrane surface and promote cake-layer development. However, the comparable fitting quality of the intermediate pore-blocking model indicates that partial penetration and deposition of oil droplets within membrane pores may also occur. Therefore, the observed fouling behavior is more appropriately described as a combination of surface cake formation and intermediate pore blocking rather than being governed exclusively by a single mechanism. The contribution of intermediate pore blocking may explain the irreversible fouling observed after hydraulic cleaning, whereas the cake layer formed on the membrane surface is expected to contribute primarily to reversible fouling. Furthermore, cake filtration typically occurs on the membrane surface and can be easily prevented by the surface cleaning step; however, pore blocking of the membrane involves specialized steps, such as backwashing or chemical cleaning, because it affects the porous structure (as schematically explained in Figure S6).

**Figure 11:**
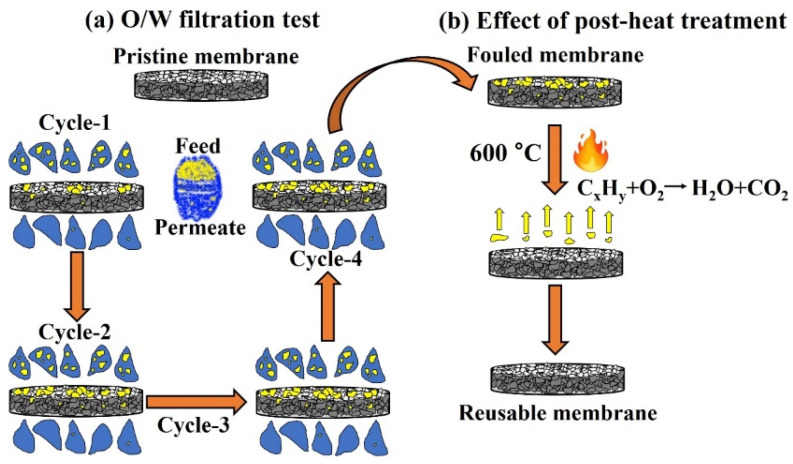
Schematics showing (**a**) O/W filtration test and membrane fouling, (**b**) removal of oil foulants from membranes by post-heat treatment.

The progressive effect of the four-cycle O/W filtration test and post-heat treatment on the fouled membrane is schematically shown in Figure 11a,b, respectively. Although the SiC showed high O/W filtration efficiency in each cycle, it was necessary to properly clean the membrane to ensure higher permeability values. Therefore, this study effectively utilizes the high thermal stability of the SiC membranes by heat treating the fouled membranes in air at temperatures ranging from 500 to 600 °C. The oily volatile foulants, mainly composed of hydrocarbon compounds, become unstable at high temperatures and hence can be completely removed from the internal pores of the membrane that are difficult to clean using the SDS solution.

**Figure 12:**
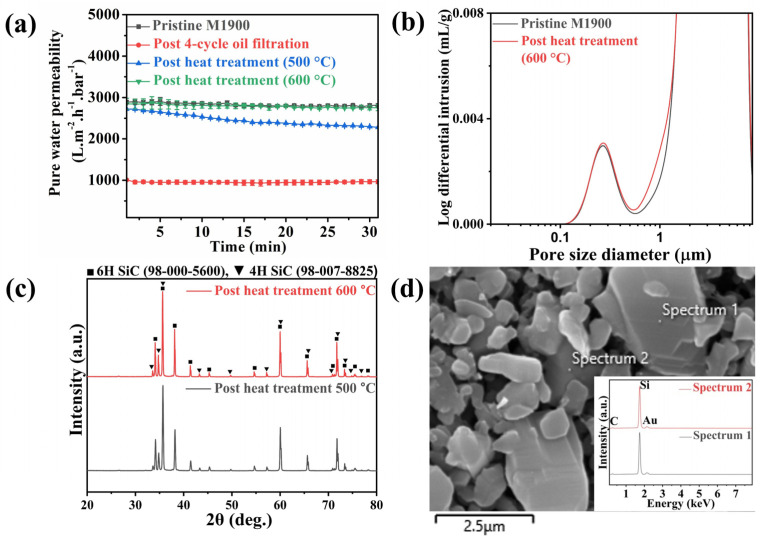
Effect of post-heat treatment on fouled membrane previously sintered at 1900 °C (M1900). (**a**) PWP comparison of pristine, fouled, and heat-treated membranes. (**b**) Pore size comparison of pristine and heat-treated membranes. (**c**) XRD result of the heat-treated membrane. (**d**) EDS spectrum of the membrane heat-treated at 600 °C.

The effect of post-O/W filtration heat treatment of the M1900 membrane on its water flux recovery capacity, pore size, and secondary phase formation is shown in Figure 12a,b and Figure 12c,d, respectively. The result showed that the heat treatment at 600 °C fully recovered the fouled M1900 membrane, with water permeability values comparable to those exhibited by the pristine M1900 membrane (Figure 12a), which was attributed to the pore size being similar to that of the pristine membrane (Figure 12b). Such a full water flux recovery highlighted that the foulants attached to the membrane could be completely removed by low-temperature heat treatment. Furthermore, the XRD (Figure 12c) and EDS (Figure 12) results of the heat-treated fouled M1900 membrane confirm the absence of a new phase, exhibiting high thermal stability and emphasizing that recrystallized SiC membranes can be repeatedly used for O/W emulsion purification. Although the thermal treatment approach is highly effective for ceramic membranes, it is inherently more energy-intensive than conventional chemical cleaning methods. Chemical cleaning typically requires lower operating temperatures and is therefore generally associated with lower direct energy consumption. However, chemical cleaning involves reagent consumption, generation of secondary waste streams, and potential long-term effects on membrane materials. In contrast, the excellent thermal stability of SiC membranes enables high-temperature regeneration without significant structural degradation. It is important to note that all filtration experiments were conducted in dead-end mode, whereas industrial oily wastewater treatment commonly utilizes cross-flow filtration. The tangential flow in cross-flow systems generates shear stress at the membrane surface, mitigating concentration polarization and cake-layer formation. As a result, higher permeate fluxes and reduced fouling rates would generally be expected compared with dead-end operation. Therefore, the flux decline and fouling behavior reported in this work may be considered a conservative assessment of membrane performance. Nevertheless, the observed trends in permeability enhancement and fouling mitigation associated with the asymmetric membrane structure are expected to remain relevant under cross-flow conditions, although the absolute values of the performance metrics may differ.

Finally, the separation performance of the fabricated SiC MF membranes was compared with that of other membranes previously reported in the literature (Table 4; [10,16,50,51,52,53,54,55,56]. Note that the performance of a membrane in O/W filtration depends on several factors, such as the size of oil droplets, composition of the emulsion, pore size of the membrane, and surface characteristics (oleophilic or oleophobic) of the membrane. The size of oil droplets and pore size of the membrane are critical parameters of pressure-driven membranes. If the size of oil droplets is much larger than the pore size of the membrane, the probability of pore blocking is relatively lower, with the mode of fouling being cake formation (membrane external fouling), which can easily be prevented by the washing step. In contrast, if the size of oil droplets is similar to the pore size of the membrane, pore blocking (membrane internal fouling) occurs during the filtration test. Nevertheless, the removal of fine oil droplets is also critical before releasing the wastewater from industries to avoid environmental pollution.

The values listed in Table 4 show that the fabricated M1900 SiC membrane demonstrated superior performance in filtering small-sized oil droplets compared to other reported membranes, exhibiting good permeability, oil rejection, and flux recovery. Previously reported studies on the filtration of oily wastewater mostly used oil emulsions with large droplets; however, this study focused on the filtration of small oil droplets from an O/W mixture. Some previously reported surface-modified composite polymeric [51,55] and fibrous Si3N4 [54] membranes exhibited good permeability and oil rejection; however, such polymeric membranes cannot be used under harsh conditions. Moreover, fibrous membranes are usually costly and difficult to manufacture on a large scale. Although conventional alumina, titania, and zirconia membranes are less expensive than SiC membranes, they exhibit a lower permeability, which is crucial for water purification on an industrial scale.

## 4. Conclusions

Asymmetric pure silicon carbide (SiC) membranes, defined by their gradient profiles, represented a robust paradigm for the efficient treatment of oil-in-water (O/W) wastewater. The asymmetric membrane structural constants (k_1_, k_2_, and k_3_) revealed that the lower hydraulic resistance (k_1_) was the dominant factor in improving its permeability as compared to its symmetric structure due to smaller membrane thickness. Despite having relatively smaller pore sizes, the fabricated asymmetric SiC membranes exhibited better water permeability as compared to their symmetric counterparts, owing to the higher values of the pore size exponent (k_2_) and improved flow pathway optimization (k_3_) attributed to their unique multilayered asymmetric structures, which closely approach the ideal pore architecture. The membranes showed an increase in PWP (1257 ± 43 LMH/bar to 3883 ± 141 LMH/bar) and O/W emulsion permeability (226 ± 15 LMH/bar to 905 ± 93 LMH/bar) with the increase in pore size (0.12–0.31 μm); however, membranes with relatively larger pore sizes exhibited a decreased separation performance (from 99% to 91%), possibly due to a decrease in the sieving effect. The M1900 membrane sintered at 1900 °C exhibited an optimal pore structure, with an average pore size of 0.26 μm and an open porosity of ~42%, resulting in a PWP of 2813 ± 40 LMH/bar, an O/W separation performance with >95% rejection, and a stable O/W emulsion permeability of ~327 LMH/bar. The M1900 SiC membrane experienced significant fouling after the O/W filtration test due to pore blocking, as indicated by the Hermia models—cake filtration and intermediate pore blocking. Notably, owing to the high thermal stability of SiC, the fouled SiC membrane could be easily regenerated to its original water flux recovery capacity by simply heating in air at 600 °C. Regeneration capability exhibited by the SiC membrane provides substantial practical advantages, allowing for long-term performance and reusability. The findings of this study provide valuable insights into the design and optimization of multilayered asymmetric ceramic membranes, offering both theoretical and experimental support for their application in O/W separation and other advanced filtration processes.

## Figures and Tables

**Table 1:** Assigned names and properties of the prepared porous SiC membranes.

Membrane Designation	Sintering Conditions (Temperature/Time/Atmosphere)	Pore Size (D_50_, μm)	Measured Pure Water Permeability (L.m^−2^.h^−1^.bar^−1^)
M1700	1700 °C/2 h/Ar	0.12	1257 ± 43
M1800	1800 °C/2 h/Ar	0.14	1545 ± 38
M1900	1900 °C/2 h/Ar	0.26	2813 ± 40
M2000	2000 °C/2 h/Ar	0.31	3883 ± 141

**Table 2:** K–C and H–P model parameters for symmetric and asymmetric SiC membranes.

Membrane Type	Modified K–C Equation			Modified H–P Equation		Refs.
	k_1_	k_2_	R^2^	k_3_	R^2^	
Ideal	1	2	-	1	-	
Symmetric	1094.270	1.596	0.828	0.074	0.980	[4]
Asymmetric	448.3	1.872	0.993	1.972	0.992	This study

**Table 3:** Parameters obtained after applying the Hermia model to the experimental data obtained from the SiC membrane sintered at 1900 °C (M1900).

Membrane Designation	Cake Filtration		Intermediate Pore Blocking		Standard Pore Blocking		Complete Pore Blocking	
	$R_{c}^{2}$	k_c_	$R_{i}^{2}$	k_i_	$R_{s}^{2}$	k_s_	$R_{b}^{2}$	k_b_
M1900	0.9805	9.9 × 10^−9^	0.9798	2.3 × 10^−5^	0.8330	8.1 × 10^−4^	0.9305	7.8 × 10^−2^

**Table 4:** Comparison of the O/W emulsion separation performance of developed SiC membrane with reported values of different ceramic membranes in the literature.

Membrane		O/W Emulsion		O/W Emulsion Permeability		Oil Rejection	Flux Recovery	Refs.
Material	Pore Size (μm)	Concentration (ppm)	Droplet size (μm)	LMH/bar	LMH	(%)	(%)	
Zirconia	0.10	100	10–40	97–120		~95%	~80	[50]
SiC	0.082	200	5–10	-	101.51–171.54	94.79 (~95)	-	[16]
SiC	0.058	200	5–10	-	69.50–99.01	96.45 (~96)	-	[16]
Alumina	0.2	250	1–10 (avg value 2)	18	-	~99	-	[10]
Alumina	0.8	250	1–10 (avg value 2)	32	-	~99	-	[10]
PES/Pluronic F127	-	1000	0.12–29.98 (avg value 2.1)	-	42.77–82.98	~100	62.07–93.33	[51]
Mullite-Alumina (0–75%)	0.289	1000	0.1–3.0 (avg value 1.09)		72.7–244	93.8–81.3	28.97–83.61	[52]
Mullite	0.29	1000	avg value 1.07	23–33	-	-	-	[53]
Mullite-Alumina (0–75%)	0.289	1000	0.3–2.4 (avg value 0.7)	-	41.3–91.5	84–70.8	46.83–96.27	[52]
Si_3_N_4_ fiber	0.680	1000	0.3–1.1 (avg value 0.680)	-	260–392	91–89	96	[54]
PVDF/TBC	0.0123	1000	0.38–0.72 (avg value 0.44)	30.25	-	99.5	77	[55]
Alumina	0.2	200–1000	-	28	-	~93	-	[56]
Titania	0.05	200–1000	-	˂5	-	~99	-	[56]
SiC	0.26	1000	0.2–0.7 (avg value 0.35)	327	-	~96%	Fouled = 78–35%, after post-heat treatment = 100%	This work

## Data Availability

The original data presented in the study are included in the article and Supplementary Materials; further inquiries can be directed to the corresponding authors.

## Supplementary Materials

The following supporting information can be downloaded at: https://www.mdpi.com/article/10.3390/membranes16060213/s1, Figure S1: Schematic depicting the droplet size distribution curve of O/W emulsion (1000 mg/L); Figure S2: Schematic depicting the fabrication of an asymmetric SiC membrane; Figure S3: Schematic showing the laboratory-scale dead-end filtration setup to measure the permeability of the fabricated SiC membranes; Figure S4: XRD patterns of SiC MF membranes sintered from 1700 to 2000 °C; Figure S5: Dynamic water contact angle (WCA) of SiC membranes sintered from 1700 to 2000 °C; Figure S6: Schematics showing the mechanism behind membrane fouling based on Hermia models; Table S1: Results of Rietveld refinement of XRD data obtained from the SiC membranes sintered from 1700 to 2000 °C.
