# Supplementary material for: The Structural Evolution of Recrystallized Asymmetric SiC Membranes for High-Performance Oily Wastewater Treatment
Source: Membranes (Basel). 2026 Jun 21;16(6):213. doi: 10.3390/membranes16060213 (PMC13304135; doi:10.3390/membranes16060213)
Supplement: Supplementary file 1 [file membranes-16-00213-s001.zip › membranes-4360874-supplementary.pdf]

Supporting Information

The Structural Evolution of Recrystallized Asymmetric SiC Membranes for High-Performance Oily Wastewater Treatment

Muhammad Shoaib Anwar, Jang-Hoon Ha, Jongman Lee, Hong Joo Lee * and In-Hyuck Song *

Nanomaterials Research Division, Korea Institute of Materials Science (KIMS), 797 Changwon-daero, Seongsan-gu, Changwon-si, Gyeongsangnam-do 51508, Republic of Korea; drmsa789@gmail.com (M.S.A.); hjhoon@kims.re.kr (J.-H.H.); jmlee@kims.re.kr (J.L.)

* Correspondence: hjlee@kims.re.kr (H.J.L.); sih1654@kims.re.kr (I.-H.S.)

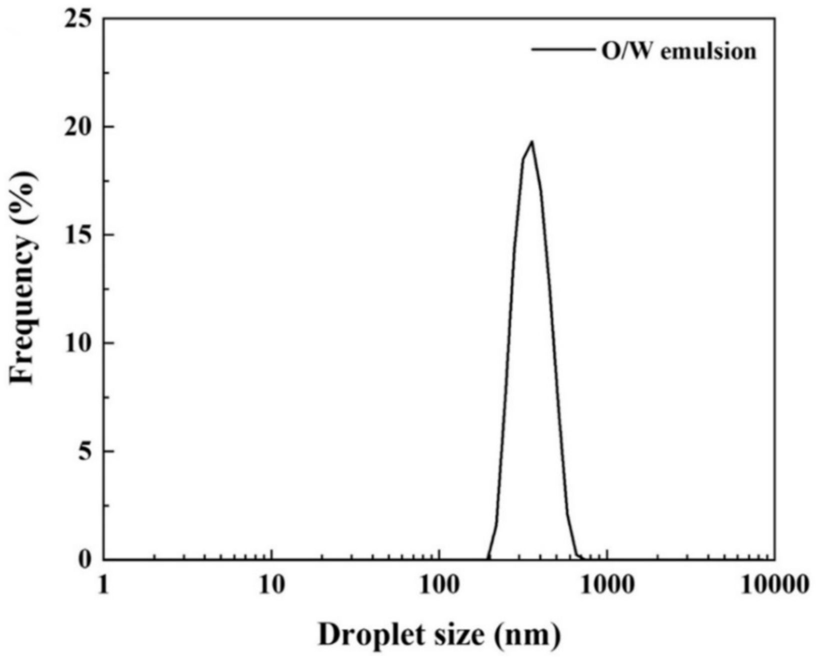

Figure S1. Droplet size distribution curve of O/W emulsion (1000 mg/L).

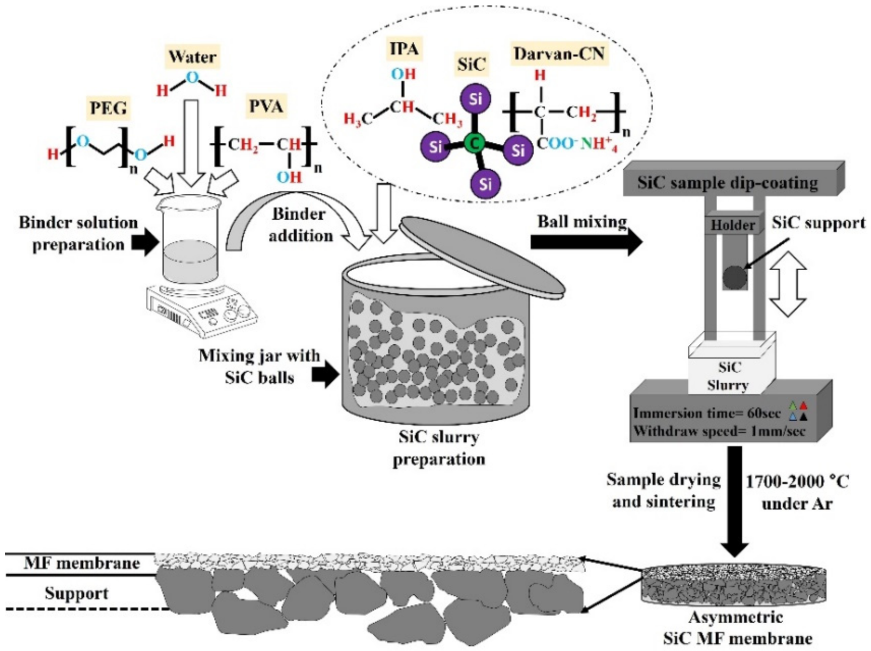

Figure S2. Schematic depicting the fabrication of an asymmetric SiC membrane.

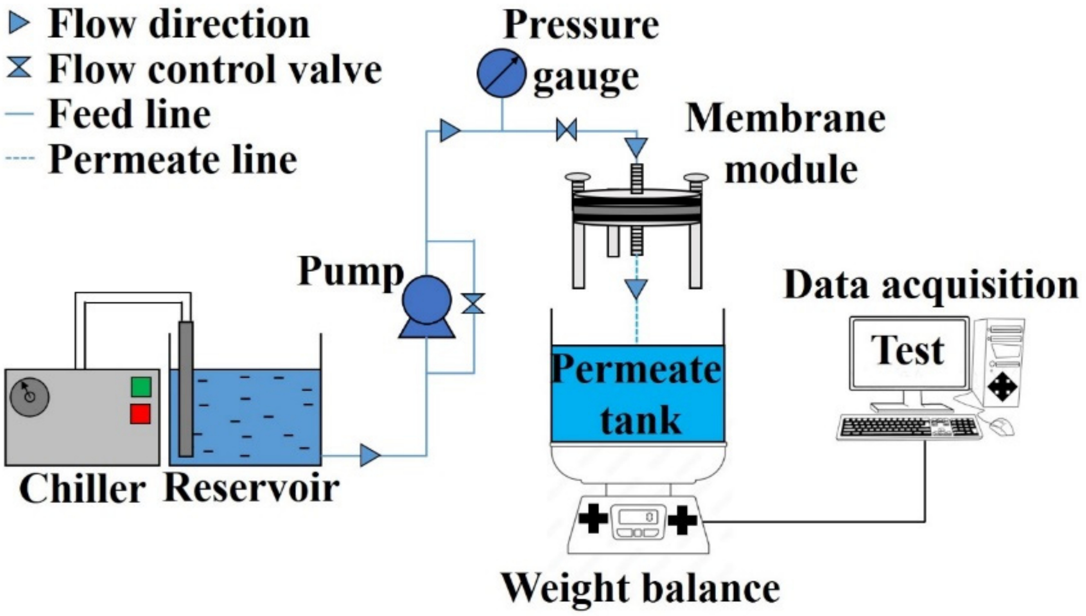

Figure S3. Schematic showing the laboratory-scale dead-end filtration set-up to measure the permeability of the fabricated SiC membranes.

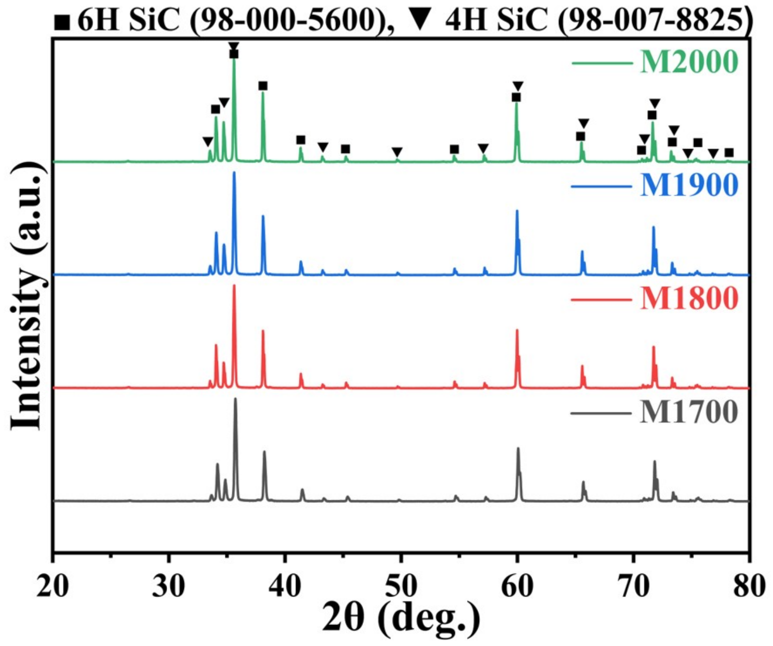

Figure S4. XRD patterns of SiC MF membranes sintered from 1700-2000 °C.

Table S1. Results of Rietveld refinement of XRD data obtained from the SiC membranes sintered from 1700-2000 °C

Membrane designation	Sintering conditions	Phase content (wt%)	
		6H-SiC	4H-SiC
M1700	1700 °C/2 h/Ar	74.9	25.1
M1800	1800 °C/2 h/Ar	74.0	26.0
M1900	1900 °C/2 h/Ar	68.3	31.7
M2000	2000 °C/2 h/Ar	64.5	35.5

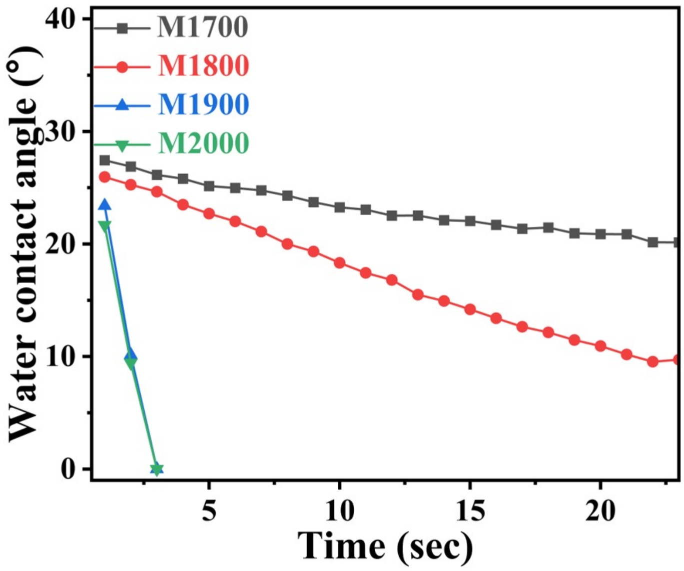

Figure S5. Dynamic water contact angle (WCA) of SiC membranes sintered from 1700-2000 °C.

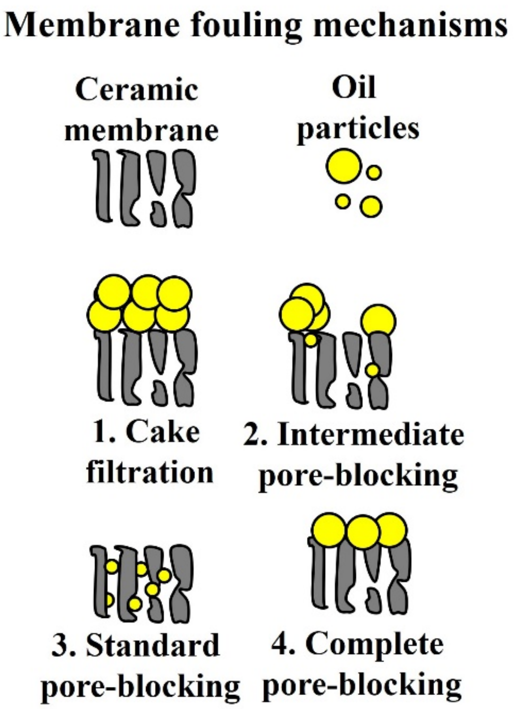

Figure S6. Schematics showing the mechanism behind membrane fouling based on Hermia models.
